# First case of systemic fatal mycobacteriosis caused by *Mycobacterium goodii* in a pet Kenyan sand boa (*Eryx colubrinus loveridgei*)

**DOI:** 10.1186/s12917-022-03351-z

**Published:** 2022-07-26

**Authors:** Alessandro Vetere, Mara Bertocchi, Teresa Bruna Pagano, Francesco Di Ianni, Giordano Nardini

**Affiliations:** 1 Clinica Veterinaria Modena Sud, Piazza dei Tintori, 1, Spilamberto, MO Italy; 2 Department of Veterinary Science, University of Parma, Strada del Taglio 10, 43126 Parma, PR Italy; 3 La Vallonea Laboratory, Via Giuseppe Sirtori, 9, 20017 Rho, MI Italy

**Keywords:** Mycobacteriosis, *Mycobacterium goodii*, *Eryx colubrinus loveridgei*, Pet snake, Reptiles, Zoonosis

## Abstract

**Background:**

Environmental nontuberculous mycobacteria species that are not members of the *M. tuberculosis* complex, are ordinary inhabitants of a wide variety of environmental reservoirs and their role in human and animal diseases has been fully recognized. Even if spontaneous mycobacterial infections have been reported in a wide variety of reptiles, this is the first report of systemic fatal mycobacteriosis sustained by *Mycobacterium goodii* in a pet reptile.

**Case presentation:**

An adult, wild caught (WC), male Kenyan sand boa (*Eryx colubrinus loveridgei*) age unknown, was presented for clinical examination due to decreased activity level, decreased appetite and diarrhea. Blood tests showed unreliable results. Coprologic exam showed a moderate to severe presence of flagellates. X rays and ultrasound showed moderate presence of air and faeces in the large intestine. The snake was hospitalized and oral metronidazole was chosen as antiprotozoal agent in association with subcutaneous warm fluids. The snake was discharged after 2 weeks therapy in good clinical condition. Faecal exam resulted negative. One month after, the snake was quickly hospitalized again because of a recrudescence of symptoms. Biochemistry showed severe increase of AST, ALT and biliary acids. Severe leucocytosis and moderate to severe anemia were highlighted. Ultrasound examination revealed a severe diffused alteration of the liver parenchyma and a fine needle aspiration was performed. The cytological diagnosis was mixed inflammation, with a numerous of unstained rod-shaped bacteria both inside macrophages and free in the sample. The snake’s condition rapidly deteriorated and euthanasia was performed. The histology of the coelomic organs confirmed a systemic mycobacteriosis. Real-time PCR identified the mycobacteria as *Mycobacterium goodii*.

**Conclusions:**

Species from the genus *Mycobacterium* are among the most important micro-organism including the causative agents of tuberculosis. Even if the general incidence of disease in reptiles due to mycobacteria is comparatively low, they can serve as reservoirs of many ubiquitous mycobacteria species. *Mycobacterium goodii* is a rapidly growing non‐tuberculous mycobacterium that has recently been associated with severe infections in animals and humans. Although in this case the pathogenesis was not completely clear, we highlight the zoonotic risk of mycobacteriosis in exotic animals especially in WC specimens.

## Background

Mycobacteria represent a very particular group of bacteria of the Actinomycetales order; they are divided into two groups known as the “*M. tuberculosis* complex” (*M. tuberculosis, M. bovis, M. microti, M. africanum, M. pinnipedii, M. caprae, *and *M. canetti*) and other mycobacteria than the “*M. tuberculosis* complex” (MOTT), also referred to as Nontuberculous mycobacteria (NTM) [1–3]. They possess capsules and most of them do not form spores [2, 4]. Strictly for clinical purposes, Mycobacteria are divided into different groups related to their characteristic growth rates in culture; the growth rate is different among the species [1, 4, 5]. Species are classified as rapid-growers and difficult-to-grow (slow-growers). Rapid-growers form mature colonies from one week to several weeks on solid culture media [1, 5]. Difficult-to-grow mycobacteria are mycobacteria that grow very slowly or do not grow at all in vitro [1, 5]. The characteristics of the Mycobacterium species is that the cell wall is hydrophobic, waxy and rich in micolic acids [6]. These slow-growing, non-motile, slender, aerobic, acid-fast, Gram-positive rods have a long history in human medicine [6]. The pathogenetic role of *Mycobacterium avium* subspecies *paratuberculosis* it is also relevant in veterinary medicine. This is the etiologic agent of paratuberculosis, a chronic contagious granulomatous enteritis characterized in cattle by persistent diarrhea, progressive weight loss, debilitation, and eventually death [7]. The infection has also been recognized in omnivorous and carnivorous mammals, such as wild rabbits, foxes, weasels, pigs, and non-human primates [7]. One theory regarding *M. avium* subsp. *paratuberculosis* (MAP) is intimately linked to the etiology of Crohn Disease (CD) in humans [8]. It is believed that due to its thick, waxy cell wall MAP is able to survive the process of pasteurization as well as chemical processes used in irrigation purification systems. Subsequently meat, dairy products and water serve as key vehicles in the transmission of MAP infection to humans who have a genetic predisposition (from farm to fork), thus leading to the development of CD [8, 9]. Spontaneous mycobacterial infections have been reported frequently in a wide variety of reptiles, like turtles, lizards, crocodiles and indeed, snakes, [10–14]. A systemic mycobacteriosis sustained by *M. thamnopheos* was diagnosed in an ill *Boa constrictor*, presented with multiple subcutaneous granulomas [15]; moreover, a dual infection sustained by *M. haemophilum* and *M. marinum* together was diagnosed in a ball python (*Python regius*) with chronic history of respiratory disease. Another two cases of mycobacteriosis with severe granulomatous pneumonia involved one exotic python snake (*Python molurus*) and one native green snake (*Philodryas olfersii*) in captivity. In both cases, *M. genavense* was implicated in the infection [16]. Mycobacterial infection appears to be most common in chelonians, compared to other Orders [17]. Affected animals are often wild-caught or free ranging animals [17, 18]. Most of mycobacterial infections are reported in sea turtles [17–19]. The affected animals showed multiple granulomatous lesions in different organs (liver, spleen, kidneys, joints and also bones) [17, 18]. In lizards there are few reports about mycobacteriosis. In one report, a granulomatous osteomyelitis was described in a pet bearded dragon (*Pogona vitticeps*) [20]. In another report a systemic mycobacteriosis was reported in a frilled lizard (*Chlamydosaurus kingii*) affected by systemic illness [21]. A mycobacterial granulomatous pneumonia was also reported in farm-raised *Crocodylus porosus, Crocodylus johnstonii* and *Crocodylus niloticus.* It is generally believed that mycobacteria are contracted through defects in the integument or by ingestion [10, 12, 18]. In reptiles, histopathologic examination shows typical granulomatous inflammations with macrophages and multinucleated giant cells [17, 18, 22]. Unlike mammalian tubercles, calcification has not been observed [10, 12, 22]. Histopathology is routinely used to diagnose mycobacteriosis in reptiles, but this technique has low sensitivity but high specificity; in histological sections, granulomatous lesions are evident on hematoxylin–eosin stains [22]. To date, new diagnostic techniques, as the Polymerase Chain Reaction (PCR) have been developed, reducing the timing needed to diagnose the disease [23]. PCR allows to detect and characterize mycobacteria using primers for the bacterial 16S ribosomal RNA gene in a few days [24–26]. Fite-Faraco and Ziehl- Neelsen acid-fast stains, are frequently used to demonstrate mycobacteria in cytological and histological samples, alone or in combination with the PCR [23]. In this case, *Mycobacterium goodii* was identified from tissue samples. *Mycobacterium goodii* is a rapidly growing non‐tuberculous mycobacterium (Runyon Group IV). In human medicine, for rapidly growing mycobacteria, antimicrobial compounds routinely tested for minimum inhibitory concentrations include amikacin, cefoxitin, ciprofloxacin, clarithromycin, doxycycline (or minocycline), imipenem, linezolid, moxifloxacin, trimethoprim-sulfamethoxazole, and tobramycin [27]; many of these bacteria are however characterized as being resistant to antibiotics and their zoonotic potential is demonstrated [28–30]. In one report, a panniculitis sustained by *M. goodii* was described in an immunocompetent, demonstrated dog, even if organisms were not detected during cytologic and histologic examinations despite multiple examinations of various tissue sections and the numerous of special stains performed [31]. In another case report, a dog was affected simultaneously by Cushing disease and nodular bacterial panniculitis sustained by *M. goodii*. In that case, the hypercortisolemia secondary to Cushing disease might have contributed to a higher risk of opportunistic mycobacterial infection [32]. A *Mycobacterium goodii*-related mastitis was also diagnosed in a 5-years old Holstein cow. In this report, poorly positive acid fast bacilli were identified in a Ziehl–Neelsen stained histological slides [33]. In the same report, authors emphasize that *M. goodii*, through contaminated milk, could be potential source for human infection [33]. *M. goodii* was also isolated in a nondomestic animal, a spotted hyena (*Crocuta crocuta)* affected by pyogranulomatous pneumonia [34]. In humans, *M. goodii* was responsible of several nosocomial infection [28, 30, 35], but no specific host risk factors have been reported. Treatment required prolonged appropriate antibiotic therapy, and often surgical debridement and contaminate material removal, in all cases of infections associated with surgical intervention or implants [28, 30, 35]. In this case, the snake owner was warned for the potential zoonotic risk of his snake and considered also the poor prognosis, agreed to euthanize his pet.

## Case presentation

**Fig. 1:**
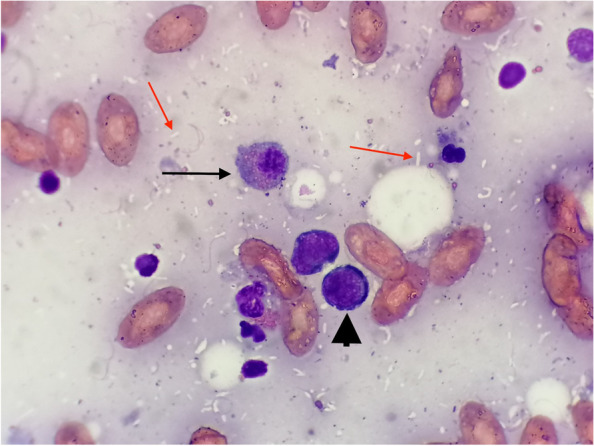
FNA of the liver of a Kenyan sand boa (*Eryx colubrinus loverdigei*). Mixed inflammation. Multiple unstained rod-shaped bacteria are visible in the sample background (red arrows). Reactive lymphocytes (arrowhead) and partially degranulated heterophils (black arrow) were numerous. Diff Quick® stain, 400x. Scale bar: 10 $\mathrm{\mu m}$

**Fig. 2:**
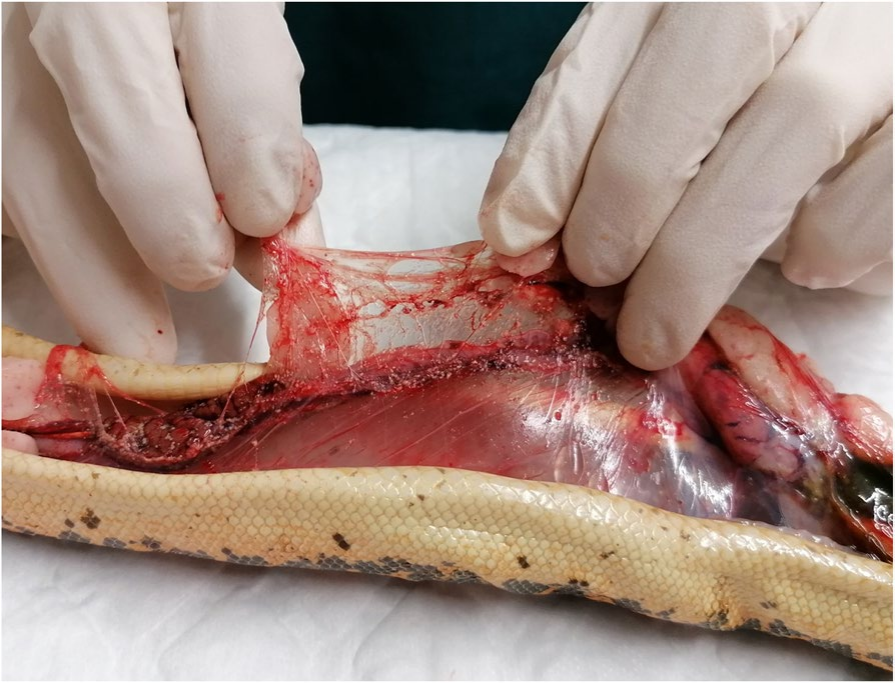
Kenyan sand boa (*Eryx colubrinus loverdigei*), necropsy. At the coelomic cavity opening, numerous miliary granulomas are visible adherents to serous membranes

**Fig. 3:**
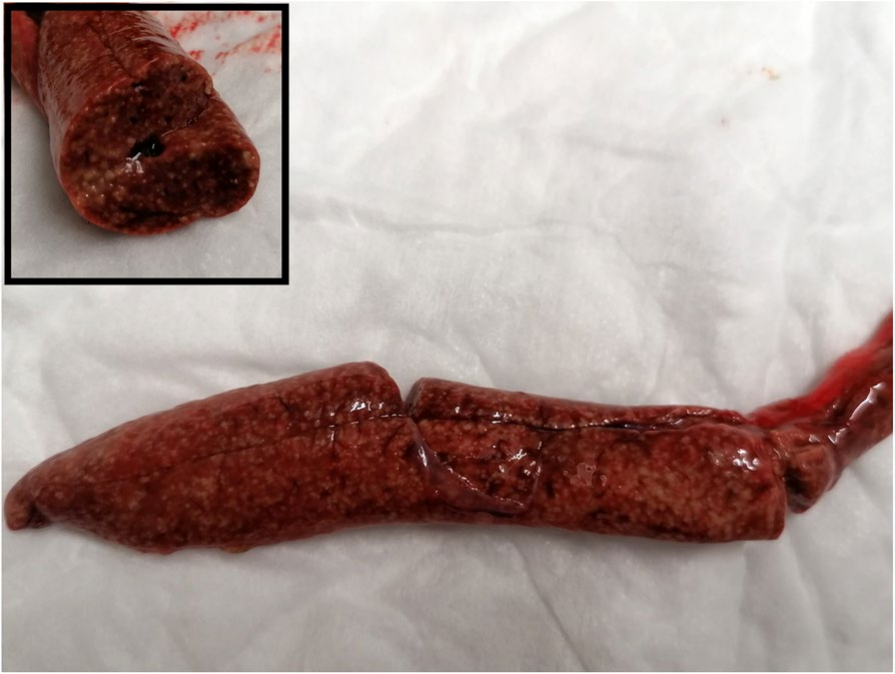
Liver of the Kenyan sand boa (*Eryx colubrinus loverdigei*), gross pathology. Multiple miliary nodules are disseminated in all the liver parenchyma

A 100 g, wild caught, adult male Kenyan sand boa (*Eryx colubrinus lovedrigei*) age unknown was presented for clinical examination due to decreased activity level, decreased appetite and diarrhea. The animal was acquired 3 months before in a reptile exhibition from a private importer; the provenience was unknown. The owner reported that the snake had only one meal one week after the day, based on a 2 g defrozen mouse, eaten without hesitation. One week after, the owner tried again with other small mice in order to evaluate its response to the offered food, but it refused to attack the mouse; after few hours, the snake had two episodes of malodorous diarrhea. The animal was housed in a glass terrarium at 32 °C (89,6°F) at day and 25 °C (77°F) at night. A UVB light 5.0 spectrum was provided. Body condition score was 4 of 5 [36] and minimal dehydration [37] (< 5%) was reported. No other alterations were evident at the physical examination. A complete blood work, faecal exam, x-rays and an ultrasound examination were performed. Biochemistry showed a mild to moderate increased of AST, ALT and ALP in comparison to the normal reference values established for *Boa constrictor*, a specie belonging to the same family (*Boidae*), since there are no reference intervals for *Eryx* sp*.* in literature to the present day (03/07/2021) (Table 1) [11, 12, 38]. The hemogram showed moderate heterophilia and azurophilia, always in comparison to *Boa constrictor* normal reference values (Table 2) [39]. A fecal fresh smear was performed, showing a moderate to severe presence of flagellates. Flotation was negative for parasites eggs. X rays and ultrasound showed only moderate presence of air and faeces in the large intestine. The snake was hospitalized for two weeks and oral metronidazole [DEFLAMON 500 mg/100 ml, Bieffe Medital S.p.A. – Via Nuova Provinciale – Grosotto (SO)] at the dosage of 50 mg/kg every 48 h for three times was chosen as antiprotozoal agent in association with subcutaneous warm fluids [Ringer solution, S.a.l.f. Spa, Via Guglielmo Marconi, 2, 24,069 Cenate sotto BG (Italy)] administered once a day. The snake was discharged after 2 weeks therapy in good clinical condition and faecal exam resulted negative after two antiprotozoal treatments. One month after the owner reported a recrudescence of symptoms. The snake was hospitalized again, and a CBC and biochemistry were repeated. Biochemistry showed severe increase of AST, ALT and ALP despite the first measurement performed one month prior (Table 3) [36]. Hematology showed severe leucocytosis and moderate to severe anemia, despite the first measurement (Table 4) [37]. Ultrasound examination was performed, revealing a severe and diffused alteration of the liver parenchyma. A fine needle aspiration (FNA) of the liver was performed. Cytology showed a mixed inflammation with a numerous of unstained rod-shaped bacteria displaying the “negative image” of mycobacteria. These organisms were evident both inside macrophages and free in the sample stained with Diff Quick® (Diff Quick Stain®, Bio Optica S.p.a, Milano, 20,100, Italia) coloration (Fig. 1). A Fite-Faraco stain was performed, confirming the acid-fast organisms. The snake’s condition was poor, and euthanasia was performed under the owner’s request. Complete necropsy was performed. At the coelomic cavity opening, multiple miliary granulomas were evident involving coelomic serous membranes (Fig. 2) liver and kidneys parenchyma and large intestine (Fig. 3). Representative tissue samples were collected from most affected organs (liver, coelomic cavity, kidneys, thyroid, large intestine), preserved in 10% neutral buffered formalin, and submitted to histopathological examination. 3 µm thick slides were stained with hematoxylin and eosin for routinary histological examination and examined by an optical microscope. Selected 3 µm thick slides were than stained with Fite-Faraco histochemical staining protocol for alcohol acid resistant bacteria. Olympus CX-43 microscope [Olympus Italia SRL, Via Modigliani 45, Segrate, 20,090, MI, Italia] equipped with Olympus EP-50 camera [EPview software, 5 Megapixel, 2592 × 1944 Resolution photograph (Pixel)] was used to capture histological and cytological images.

**Table 1:** Comparison between biochemical values of the patient (*Eryx colubrinus loveridgei*) and the reference values for *Boa constrictor *

	Parameters	Reference Values
AST (U/l)	366	3 – 331
ALP	**1444**	43–1342
Total protein (g/l)	26	24 – 48
Albumin (g/l)	10	7.8 – 17.5
Creatinkinasis (U/l)	985	53–1328
LDH (U/l)	270	16 – 877
Phosphorous (mmol/l)	2,8	2,6 – 11,7
Calcium (mmol/l)	13,6	13,5 – 16,2
Potassium (mmol/l)	6,0	3,3 – 11,2
ALT (U/l)	**321**	8–132

^a^[38]

**Table 2:** Comparison between CBC (Complete Blood Count) parameters of the patient and the reference values

	Prameters	Reference values^a^
WBC × 10^3^/mm^3^	**40,2**	0,88 – 22,6
RBC × 10^6^/mm^3^	2,4	0,16–2,1
Heterophils (10^3^/µL)	**19**	0,21–12,3
Eosinophils (10^3^/µL)	0	0,03–1,22
Basophils (10^3^/µL)	0	0,03–2,77
Azurophils (10^3^/µL)	**17**	0,02–6,55
Lymphocytes (10^3^/µL)	**11**	0,16–18,05

^a^[39]

**Table 3:** Comparison between biochemical values of the patient and the reference values, one month later

	Parameters	Reference Values ^a^
AST (U/l)	**790**	3 – 331
ALP	**1688**	43–1342
Total protein (g/l)	29	24 – 48
Albumin (g/l)	8	7.8 – 17.5
Creatinkinasis (U/l)	77	53–1328
LDH (U/l)	331	16 – 877
Phosphorous (mmol/l)	2,9	2,6 – 11,7
Calcium (mmol/l)	15,5	13,5 – 16,2
Potassium (mmol/l)	3,6	3,3 – 11,2
ALT (U/l)	560	8–132

^a^[38]

**Table 4:** Comparison between CBC (Complete Blood Count) parameters of the patient and the reference values, one month later

	Parameters	Normal values^a^
WBC × 10^3^/mm^3^	**46,2**	0,88 – 22,6
RBC × 10^6^/mm^3^	**0,09**	0,16–2,1
Heterophils (10^3^/µL)	**22**	0,21–12,3
Eosinophils (10^3^/µL)	0	0,03–1,22
Basophils (10^3^/µL)	0	0,03–2,77
Azurophils (10^3^/µL)	**20**	0,02–6,55
Lymphocytes (10^3^/µL)	17	0,16–18,05

^a^[39]

**Fig. 4:**
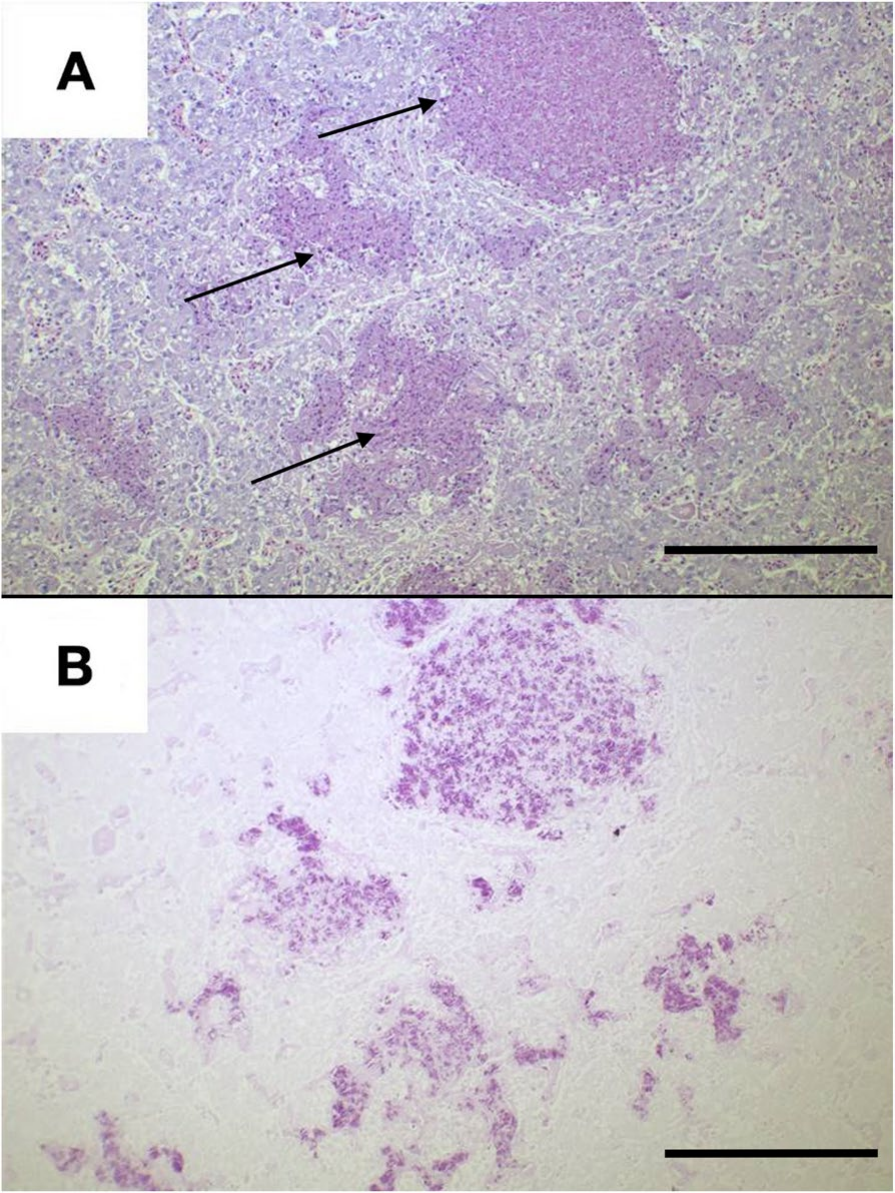
**A** Liver Kenyan sand boa (*Eryx colubrinus loverdigei*), 10X, Hematoxylin and Eosin stain. Multiple granulomas with central necrosis (black arrows). **B** Liver, 10X, Fite-Faraco stain. Many aggregates of positive red bacilli within granuloma’s centers. Scale bar: 200 $\mathrm{\mu m}$

Histopathologically, liver, kidneys and serous membranes revealed multiple, disseminated, up to 200 µm granulomas with central coagulative and colliquative necrosis (Fig. 4 A). Many vacuolated macrophages contained unstained rod-like structures. The same rod-like structure were markedly positive (red) with a Fite Faraco stain, consistently with acid fast bacteria (Fig. 4 B). A molecular exam (real-time PCR) was then performed on formalin fixed tissue sections in order to type the mycobacteria, using the Primerdesign genesig Kit (Primerdesign Ltd,Unit 1 Watchmoor Point, Watchmoor Road, Camberley, GU15 3AD) for *Mycobacterium* (all species). Bacterial DNA was extracted using QIAsymphony SP (Quiagen Italia, Via Filippo Sassetti, 16, 20,124 Milano MI) automatic extractor and purified using QIAgen DSP virus/pathogen mini kit (Quiagen Italia, Via Filippo Sassetti, 16, 20,124 Milano MI). The hypervariable region A of the 16S rRNA gene was amplified from the extracted DNA using PyroMark PCR kit (QIAGEN) and MOTT 16S primers for PCR (QIAGEN), forward primer 5’-AGTTTGATCMTGGCTCAG-3’ and reverse primer 5’-GGACTACHAGGGTATCTAAT-3’. The resulting BLAST search (https://blast.ncbi.nlm.nih.gov) confirmed 100% homology to *Mycobacterium goodii.*

## Discussion and conclusions

There are several theories about the pathogenesis of most of the mycobacteria: in humans, is demonstrated that mycobacteria inhibit the maturation of phagocytosis by suppressing the acidification of phagosomes and then they persist in the relatively lower acidic environment (pH ~ 6.2) [30]. Moreover, some proteins secreted by strongly virulent *Mycobacterium tuberculosis*, such as superoxide dismutase, hydrogen peroxide/peroxidase KatG, serine threonine protein kinase PknE, I type NADH dehydrogenase NuoG, Rv3654c and Rv3655c inhibit macrophage apoptosis [38, 39]. In reptiles, these mechanisms are not completely clear. There does not appear to be any particular species susceptibility to mycobacteriosis. In both human and animals, individual susceptibility does exist and is believed to be related to diminished host immune response [6, 16]. The infection is likely to be acquired by the ingestion of infected food or via defects in the integumentary, respiratory, urogenital systems [6, 10, 11]. In reptiles, mycobacteriosis has proved to be a not uncommon disease, but due to the difficulty of bacterial isolation and the low sensitivity of some histological staining methods, it is possible that many cases were undiagnosed [17]. Captive reptiles are often kept in suboptimal conditions, circumstances that can suppress their immune system. Many pathologies are often related to incorrect husbandry and a subsequent immune suppression [16, 40] and even if immunodeficiency is a clinical condition hard to assess in reptiles, stress could be an important predisposing factor for the developing of systemic mycobacteriosis. Hematologic evaluation provided useful indicators of chronic inflammatory disease, but none were considered specific for mycobacteria infection. Clinical suspicion of mycobacteriosis relies on detection of acid-fast organisms in fine-needle aspirates, coelomic fluids, exudates or tissue biopsy specimens [10, 11]. In this case, the identification of acid-fast bacilli in the cytologic samples, made the suspicious for systemic mycobacteriosis, then confirmed at the histological examination by a real-time PCR performed on formalin fixed tissue sections. To the best of our knowledge, this is the first report of systemic mycobacteriosis sustained by *Mycobacterium goodii* in a reptile, especially in a pet snake. The zoonotic potential of *M. goodii* has also been documented [29, 30]. Septic arthritis and osteomyelitis are restricted to immunocompromised patients (e.g., organ transplant recipients or those concurrently infected with human immunodeficiency virus) [29, 30]. Reptiles are often household pets, and, if infected, they can be a source of pathogens for the owners. The risk for humans is higher when the infected animals do not show clinical signs, because they are not treated. Although in this case the pathogenesis was not completely clear, we highlight the zoonotic risk of mycobacteriosis in exotic animals especially in WC specimens, due to their presence in the soil and aquatic environments, and their ability to grow in ectotherms, such as reptiles [17, 41]. Despite the lesions observed in this case, cold-blooded animals are considered naturally resistant to mycobacteria, because they often harbor these microorganisms without showing any symptoms [17]. The general incidence of disease in reptiles due to mycobacteria is comparatively low, but they can serve as reservoirs of many ubiquitous mycobacteria species [42]. In most cases of reptile mycobacteriosis, treatment is not advised because of the chronic and often advanced stage of the disease, long-term and expensive nature of potential treatment regimens and the potential risk of spread to other animals as well as humans [43, 44]. Furthermore, no successful treatment regimen has yet been reported for reptiles [11]. Therefore, euthanasia is generally recommended. 

## Data Availability

All data generated or analyzed during this study are included in this published article [and its supplementary information files].

## Abbreviations

°C: Celsius degrees
°F: Fahrenheit degrees
AST: Aspartate aminotransferase
ALT: Alanine aminotransferase
CBC: Complete blood count
FNA: Fine needle aspiration
Hct: Hematocrit
LDH: Lactic Acid Dehydrogenase
ALP: Alkaline Phosphatase
RBC: Red blood cells
SC: Subcutaneous
WBC: White blood cells
WC: Wild Caught
